# A Bio-Inspired Visual Neural Model for Robustly and Steadily Detecting Motion Directions of Translating Objects Against Variable Contrast in the Figure-Ground and Noise Interference

**DOI:** 10.3390/biomimetics10010051

**Published:** 2025-01-14

**Authors:** Sheng Zhang, Ke Li, Zhonghua Luo, Mengxi Xu, Shengnan Zheng

**Affiliations:** 1 College of Information Science and Engineering, Hohai University, Nanjing 211100, China; shengzhanghh@hhu.edu.cn (S.Z.); zhengsn@njit.edu.cn (S.Z.); 2 School of Mechanical and Electrical Engineering, Nanchang Institute of Technology, Nanchang 330044, China; 3 School of Computer Engineering, Nanjing Institute of Technology, Nanjing 211167, China; mengxixunjit@gmail.com

**Keywords:** Drosophila, LPTC neurons, motion directions, translating objects, variable contrast in figure-ground, environmental noise interference

## Abstract

(1) Background: At present, the bio-inspired visual neural models have made significant achievements in detecting the motion direction of the translating object. Variable contrast in the figure-ground and environmental noise interference, however, have a strong influence on the existing model. The responses of the lobula plate tangential cell (LPTC) neurons of Drosophila are robust and stable in the face of variable contrast in the figure-ground and environmental noise interference, which provides an excellent paradigm for addressing these challenges. (2) Methods: To resolve these challenges, we propose a bio-inspired visual neural model, which consists of four stages. Firstly, the photoreceptors (R1–R6) are utilized to perceive the change in luminance. Secondly, the change in luminance is divided into parallel ON and OFF pathways based on the lamina monopolar cell (LMC), and the spatial denoising and the spatio-temporal lateral inhibition (LI) mechanisms can suppress environmental noise and improve motion boundaries, respectively. Thirdly, the non-linear instantaneous feedback mechanism in divisive contrast normalization is adopted to reduce local contrast sensitivity; further, the parallel ON and OFF contrast pathways are activated. Finally, the parallel motion and contrast pathways converge on the LPTC in the lobula complex. (3) Results: By comparing numerous experimental simulations with state-of-the-art (SotA) bio-inspired models, we can draw four conclusions. Firstly, the effectiveness of the contrast neural computation and the spatial denoising mechanism is verified by the ablation study. Secondly, this model can robustly detect the motion direction of the translating object against variable contrast in the figure-ground and environmental noise interference. Specifically, the average detection success rate of the proposed bio-inspired model under the pure and real-world complex noise datasets was increased by 5.38% and 5.30%. Thirdly, this model can effectively reduce the fluctuation in this model response against variable contrast in the figure-ground and environmental noise interference, which shows the stability of this model; specifically, the average inter-quartile range of the coefficient of variation in the proposed bio-inspired model under the pure and real-world complex noise datasets was reduced by 38.77% and 47.84%, respectively. The average decline ratio of the sum of the coefficient of variation in the proposed bio-inspired model under the pure and real-world complex noise datasets was 57.03% and 67.47%, respectively. Finally, the robustness and stability of this model are further verified by comparing other early visual pre-processing mechanisms and engineering denoising methods. (4) Conclusions: This model can robustly and steadily detect the motion direction of the translating object under variable contrast in the figure-ground and environmental noise interference.

## 1. Introduction

In nature, Drosophila, as a model organism, has evolved over millions of years to develop a robust and stable visual neural system that plays a vital role in its survival, and it has better motion sensitivity than humans [1,2]. Detecting the motion direction of the translating object has become one of the fundamental survival skills of Drosophila, which plays a significant role in daily behavior, such as avoiding predators, chasing prey, and so forth [3,4]. A variety of bio-inspired models are proposed based on motion-sensitive visual neurons of Drosophila [5], which are widely used in robots [6] and drones [7]. From biology to bio-inspired modeling and even to biomimetic applications, the physiological mechanisms of the Drosophila visual neural system provide many inspirations. The motion direction of the object, as one of the important motion features of the object, provides a basis for the higher-order neural structure (central brain) to make decisions [8]. The initial motion-directional detection model to express the behavioral and physiological characteristics of Drosophila is the elementary motion detector (EMD) [9], which is widely used in the fields of intelligent devices [10,11]. However, the detection performance of the EMD and a series of improved EMD-based models [12,13,14] under complex dynamic backgrounds is not satisfactory. With the revelation of the physiological mechanism of the motion-sensitive neurons of Drosophila, the LPTC has been shown to have a strong response to translating wide-field objects and the motion of local salient objects [15]. Subsequently, several bio-inspired models based on the LPTC have been put forward by mathematicians, which have proven to have excellent detection performance under complex dynamic backgrounds [16]. In the visual neural system of Drosophila, the retina and the optic lobe are two significant biological structures for detecting motion features and objects. The retina is responsible for perceiving the change in luminance and color information from the natural environment, while the optic lobe is a multi-layered structure responsible for processing the change in luminance and color information, and further detecting motion features and objects [17,18].

The visual neural system of Drosophila is a visual invariant, highly parallel, and multi-layer ganglion information processing model. The first-order visual neural structure is the retina, which consists of approximately 800 ommatidia in each ommateum. Each ommatidium consists of eight different photoreceptor cells, R1–R8, of which R1–R6 are responsible for perceiving a wide range of light and motion information, while R7 and R8 are responsible for perceiving the color information [19]. The second-order higher visual neural structure is the optic lobe, which includes the lamina, the medulla, and the lobula complex (including lobula and lobula plate). Firstly, in the lamina, a large number of the LMC neurons receive the signal from the R1–R6 neurons, which are highly sensitive to the change in luminance [20]. The LMC neurons mainly include two types, namely, L1 and L2. The L1 type of the LMC has a strong response to increased luminance, while the L2 type of the LMC has a strong response to decreased luminance [21]. Further, the inter-neurons in the lamina have the property of improving environmental disturbance [22] and the characteristic of the LI biological mechanism, which can improve motion boundaries [23]. Secondly, a great number of the transmedulla 3, 2, and 1 (Tm3, Tm2, and Tm1) and intrinsic medulla 1 (Mi1) neurons in the medulla receive the signal from the LMC. The Tm3 and Mi1 neurons are highly sensitive to increased luminance with the response of the Mi1 delayed relative to the Tm3, on the contrary, the Tm2 and Tm1 neurons are highly sensitive to decreased luminance with the response of the Tm1 delayed relative to the Tm2 [24]. Thirdly, the T4 neurons in the medulla have four sub-types, which are highly sensitive to increased luminance and receive signals from the Tm3 and Mi1 neurons [25]; additionally, the ON/OFF contrast pathways will be activated in the medulla [26]. Finally, the T5 neurons in the lobula of the lobula complex have four sub-types, which are highly sensitive to decreased luminance and receive signals from the Tm2 and Tm1 neurons [27]; after that, the LPTC and the lobula plate-intrinsic (LPi) neurons in the lobula plate of the lobula complex [28,29,30] are adopted to detect the motion direction of the translating object. Although the Drosophila-inspired visual neural model based on the visual neural circuits has made remarkable progress in detecting the motion direction of the translating object, the detection performance is not satisfactory in the case of visual contrast in the figure-ground and environmental noise interference. Furthermore, there is scant literature that comprehensively analyses and verifies this. This study proposes a bio-inspired visual neural model to resolve these challenges. In summary, the main contributions of this paper are as follows:

(1) We utilize the spatio-temporal biological mechanism to suppress environmental noise and improve motion boundaries, which facilitate the subsequent detection of the motion direction of the translating object.
(2) We adopt the contrast normalization mechanism and the contrast pathways to reduce the fluctuation in the response of variable contrast in the figure-ground.
(3) The computing of Drosophila’s four-layer visual neural circuits demonstrates a compact and layered solution to robustly and steadily detect the motion direction of the translating object against variable contrast in the figure-ground and noise interference, which further approximates Drosophila’s visual neural detection capability.

The remainder of this paper is organized as follows. In Section 2, the related works are reviewed. In Section 3, the proposed bio-inspired model is introduced in detail. In Section 4, the experimental simulations and performance are analyzed based on the synthetic noisy visual stimulus sequence dataset. In Section 5, the proposed bio-inspired model is discussed and prospects for future research are presented. In Section 6, the proposed bio-inspired model is summarized.

## 2. Related Works

Over the years, numerous works based on the visual neural system of Drosophila have been presented. Some of the notable ones, including spatio-temporal biological mechanisms, bio-inspired motion direction detection models, and contrast neural computation, are discussed below.

### 2.1. Spatio-Temporal Biological Mechanisms

The spatio-temporal biological mechanisms work together to play a significant role in reducing background interference, which facilitates the subsequent detection of the motion direction of the translating object. The spatio-temporal biological mechanism has been shown to suppress environmental noise and improve motion boundaries, which is considered a suitable filter prior to motion and contrast information detection in the visual neural system of Drosophila [16,22]. The suitable filter mainly includes the spatial denoising mechanism [31] inspired by grouping-layer processing mechanisms [32] and the role of lateral excitation [33], and the spatio-temporal LI mechanism inspired by the biological neural system, i.e., the inhibition that occurs between the adjacent neurons [23]. The review of relevant literature is shown in Table 1.

### 2.2. Bio-Inspired Motion Direction Detection Models

To further incorporate the latest biological findings of the motion-sensitive neural mechanisms, the EMD model [9] has been optimized and several optimized models have been presented; additionally, according to the physiological mechanism of LPTC neurons, several LPTC-based bio-inspired models were presented. The relevant literature reviews are shown in Table 2.

### 2.3. Contrast Neural Computation

To adapt to the highly variable natural environment, some related researchers proposed the environmental statistics method described earlier; nevertheless, the challenge of highly variable inputs had not been fully resolved [40,41]. Through the latest relevant investigations, contrast visual computation is presented to resolve this challenge, which includes the contrast normalization mechanism and the parallel contrast pathways; the relevant literature reviews are shown in Table 3. According to the relevant literature, this mechanism has not yet been verified in detecting the motion direction of the translating object.

## 3. Methods

**Figure 1:**
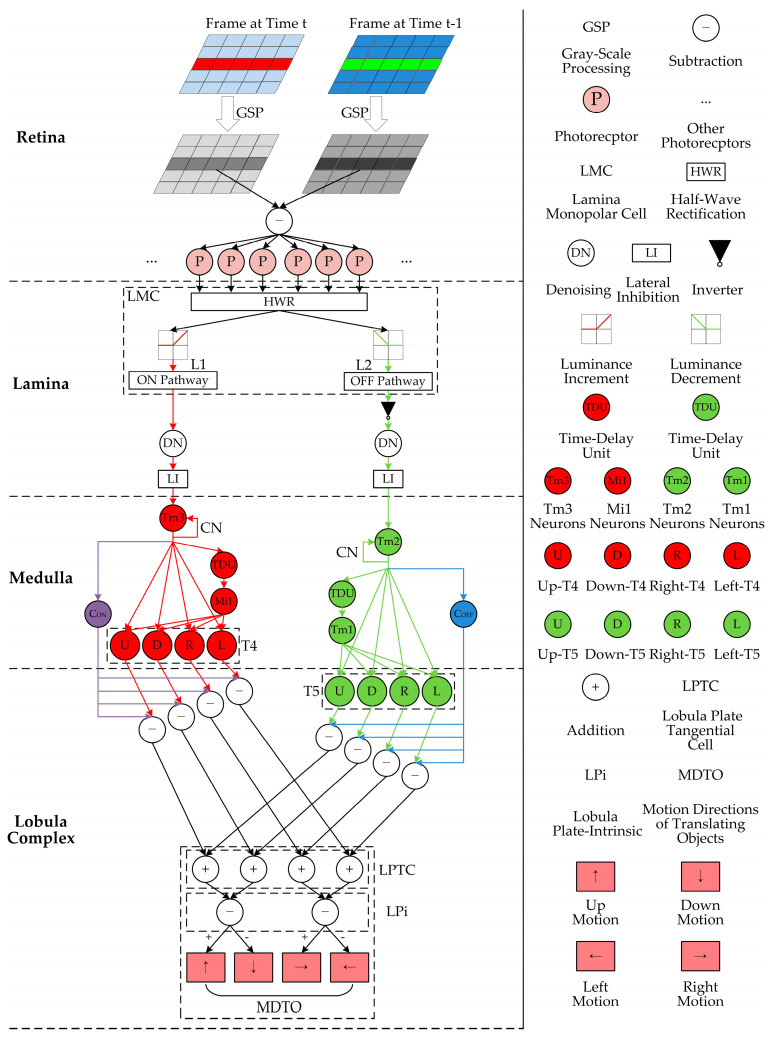
Network structure and legend of the proposed bio-inspired model: the red and green pipelines represent ON and OFF motion pathways, respectively; the purple and blue pipelines represent ON and OFF contrast pathways, respectively.

The proposed bio-inspired model is developed with formulation on four-layer neurons including the retina, lamina, medulla, and lobula complex, together with the model parameter configuration. Figure 1 depicts the network structure and legend of the proposed bio-inspired model, which consists of four-layer neurons. Firstly, the change in luminance is perceived by photoreceptors (R1–R6) in the retina neural layer. Secondly, the change in luminance is divided into parallel ON and OFF pathways by the LMC; the spatial denoising mechanism is adopted to suppress environmental noise; and the spatio-temporal LI mechanism is utilized to improve motion boundaries in the lamina neural layer. Thirdly, the ON and OFF signals are normalized and the parallel ON and OFF contrast pathways are activated in the medulla neural layer. Finally, the contrast and motion pathways converge on the LPTC in the lobula-complex neural layer to detect the motion direction of the translating object.

### 3.1. Retina Neural Layer

In the retina neural layer, Drosophila has a compound eye structure, each compound eye includes numerous ommatidia, each ommatidium consists of several photoreceptors, and each photoreceptor corresponds to a pixel [47]. These photo- receptors (R1–R6) are able to perceive the change in luminance frame-by-frame and transmit it to downstream neurons for further processing. Specifically, this model adopts gray-scale processing [48,49] and the difference between two successive frames [16] to obtain the change in luminance (see GSP, ㊀, and P in the retina neural layer, shown in Figure 1).

### 3.2. Lamina Neural Layer

In the lamina neural layer, a large number of LMC neurons (L1 and L2) (see the dashed rectangular box LMC in the lamina neural layer shown in Figure 1) separate the change in luminance into parallel ON (L1) and OFF (L2) pathways, which encode increased and decreased luminance, respectively. The half-wave rectification (HWR) algorithm is used to divide the change in luminance into the ON and OFF pathways [43] (see HWR, ON Pathway, and OFF Pathway in the lamina neural layer shown in Figure 1). The ON signal corresponds to the L1 type of the LMC, while the OFF signal corresponds to the L2 type of the LMC, and the OFF signal is inverted (see ▼ in the lamina neural layer shown in Figure 1).

Afterward, the spatial denoising mechanism is used to suppress environmental noise, which includes two stages. In the first stage, the value of the passing coefficient is computed, which is defined in Equations (1) and (2):

(1) $${\mathrm{ALC}}_{\mathrm{ON}/\mathrm{OFF}}\left(x,y,t\right)=\sum_{u=-1}^{1}\sum_{v=-1}^{1}L_{\mathrm{ON}/\mathrm{OFF}}\left(x+u,y+v,t\right)\cdot W_{\mathrm{ON}/\mathrm{OFF}}\left(u+1,v+1\right),$$

(2) $$\left[W_{\mathrm{ON}/OFF}\right]=\left[\begin{array}{l} 1/9\;1/9\;1/9\\ 1/9\;1/9\;1/9\\ 1/9\;1/9\;1/9 \end{array}\right],$$

where ${\mathrm{ALC}}_{\mathrm{ON}/\mathrm{OFF}}\left(x,y,t\right)$ represents the average luminance change in ON and OFF signals, which represents the passing coefficient; $L_{\mathrm{ON}/\mathrm{OFF}}\left(x,y,t\right)$ represents the ON and OFF signals; and $\left[W_{\mathrm{ON}/OFF}\right]$ represents the weighting matrix of ON and OFF signals. The cluster excitation obtains a larger passing coefficient, while the isolated excitation obtains a smaller passing coefficient. Then, the normalized passing coefficient is calculated, which is defined in Equation (3):

(3) $${\mathrm{NPC}}_{\mathrm{ON}/\mathrm{OFF}}\left(x,y,t\right)={\mathrm{ALC}}_{\mathrm{ON}/\mathrm{OFF}}\left(x+u,y+v,t\right)/\left(\Delta c+\max\left({\mathrm{ALC}}_{\mathrm{ON}/\mathrm{OFF}}\right)\right),$$

where ${\mathrm{NPC}}_{\mathrm{ON}/\mathrm{OFF}}\left(x,y,t\right)$ represents the normalized passing coefficient of ON and OFF signals and Δc represents a small real number to prevent the denominator tending toward zero. In the second stage, the value of the signal is multiplied by the corresponding normalized passing coefficient and then transmitted, as defined in Equation (4):

(4) $${\mathrm{DN}}_{\mathrm{ON}/\mathrm{OFF}}\left(x,y,t\right)=L_{\mathrm{ON}/\mathrm{OFF}}\left(x,y,t\right)\cdot NPC_{\mathrm{ON}/\mathrm{OFF}}\left(x,y,t\right),$$

where ${\mathrm{DN}}_{\mathrm{ON}/\mathrm{OFF}}\left(x,y,t\right)$ represents the ON and OFF signals after the denoising. Additionally, the threshold comparison is adopted to filter the decayed excitation, i.e., the isolated excitation.

Finally, each inter-neuron in the lamina neural layer receives the LI from its adjacent similar neurons, which can be achieved by the delayed propagation mode, and the relevant references verify its validity [33,35] (see LI in the lamina neural layer shown in Figure 1).

### 3.3. Medulla Neural Layer

In the medulla neural layer, the inter-neurons (Tm3, Tm2, Mi1, and Tm1) receive the ON and OFF signals, and the contrast normalization is calculated by a hyperbolic tangent tanh function (see Tm3, Tm2, and CN in the medulla neural layer shown in Figure 1), which is defined in Equation (5):

(5) $$N_{\mathrm{ON}/\mathrm{OFF}}\left(x,y,t\right)=\tanh\left(\frac{S_{\mathrm{ON}/\mathrm{OFF}}\left(x,y,t\right)}{{\hat{S}}_{\mathrm{ON}/\mathrm{OFF}}\left(x,y,t\right)+\psi }\right),$$

where $N_{\mathrm{ON}/\mathrm{OFF}}\left(x,y,t\right)$ represents the normalized ON and OFF signals, $S_{\mathrm{ON}/\mathrm{OFF}}\left(x,y,t\right)$ represents the ON and OFF signals after the early visual pre-processing, ψ represents the baseline contrast sensitivity, and ${\hat{S}}_{\mathrm{ON}/\mathrm{OFF}}\left(x,y,t\right)$ represents ON and OFF signals after the convolution, which are defined in Equations (6) and (7):

(6) $${\hat{S}}_{\mathrm{ON}/\mathrm{OFF}}\left(x,y,t\right)=\sum_{u=-5}^{5}\sum_{v=-5}^{5}S_{\mathrm{ON}/\mathrm{OFF}}\left(x+u,y+v,t\right)\cdot G_{\sigma }\left(u+5,v+5\right),$$

(7) $$G_{\sigma }\left(u,v\right)=\frac{1}{2\pi \sigma^{2}}\exp\left(-\frac{u^{2}+v^{2}}{2\sigma^{2}}\right),$$

where σ represents the standard deviation and $G_{\sigma }\left(u,v\right)$ represents a two-dimensional Gaussian kernel. Furthermore, the medulla neural layer again splits the signals into parallel contrast and motion pathways. The local contrast signal is obtained by calculating the competition between the response of the central and the surrounding adjacent neurons (see C_ON_ and C_OFF_ in the medulla neural layer shown in Figure 1), which are defined in Equations (8) and (9):

(8) $$C_{\mathrm{ON}/\mathrm{OFF}}\left(x,y,t\right)=\left|N_{\mathrm{ON}/\mathrm{OFF}}\left(x,y,t\right)-\sum_{u=-1}^{1}\sum_{v=-1}^{1}N_{\mathrm{ON}/\mathrm{OFF}}\left(x+u,y+v,t\right)\cdot W_{1}\left(u+1,v+1\right)\right|,$$

(9) $$\left[W_{1}\right]=\left[\begin{array}{l} 1/8\;1/8\;1/8\\ 1/8\;0\;1/8\\ 1/8\;1/8\;1/8 \end{array}\right],$$

where $C_{\mathrm{ON}/\mathrm{OFF}}\left(x,y,t\right)$ represents the local contrast signals of the ON and OFF pathways (see purple C_ON_ and blue C_OFF_ in the medulla neural layer of Figure 1) and $\left[W_{1}\right]$ represents the weighting matrix of the center-surrounding competition.

Next, the T4 neurons have integrated signals from the Tm3 and Mi1 neurons. At the same time, the T4 neurons have four sub-types, each of which is sensitive to one of the upward, downward, leftward, and rightward motion directions, i.e., have four different direction-selective responses. This model adopts the computing between the current and delayed normalized ON signals to separate the ON pathway into upward, downward, rightward, and leftward sub-visual pathways. The calculation formula of the separating operation of the T4 is defined in Equation 10:

(10) $$\left\{\begin{array}{l} T4_{U}\left(x,y,t\right)=N_{ON}\left(x,y-\mathrm{sd},t\right)\cdot N_{\mathrm{ON}}^{D}\left(x,y,t\right)-N_{ON}\left(x,y,t\right)\cdot N_{\mathrm{ON}}^{D}\left(x,y-\mathrm{sd},t\right)\\ T4_{D}\left(x,y,t\right)=N_{ON}\left(x,y+\mathrm{sd},t\right)\cdot N_{\mathrm{ON}}^{D}\left(x,y,t\right)-N_{ON}\left(x,y,t\right)\cdot N_{\mathrm{ON}}^{D}\left(x,y+\mathrm{sd},t\right)\\ T4_{R}\left(x,y,t\right)=N_{ON}\left(x+\mathrm{sd},y,t\right)\cdot N_{\mathrm{ON}}^{D}\left(x,y,t\right)-N_{ON}\left(x,y,t\right)\cdot N_{\mathrm{ON}}^{D}\left(x+\mathrm{sd},y,t\right)\\ T4_{L}\left(x,y,t\right)=N_{ON}\left(x-\mathrm{sd},y,t\right)\cdot N_{\mathrm{ON}}^{D}\left(x,y,t\right)-N_{ON}\left(x,y,t\right)\cdot N_{\mathrm{ON}}^{D}\left(x-\mathrm{sd},y,t\right) \end{array}\right.,$$

where $T4_{U}\left(x,y,t\right)$, $T4_{D}\left(x,y,t\right)$, $T4_{R}\left(x,y,t\right)$, and $T4_{L}\left(x,y,t\right)$ represent the signal of upward, downward, rightward, and leftward sub-visual pathways of T4 (see U, D, R, and L of the rectangular dotted box T4 in the medulla neural layer shown in Figure 1); $N_{\mathrm{ON}}^{D}\left(x,y,t\right)$ represents the delayed normalized ON signal, which can be obtained by using the temporally delayed output from Tm3 [16,43] (see red TDU and Mi1 in the medulla neural layer shown in Figure 1); and sd represents the sampling distance in every pairwise detector.

### 3.4. Lobula-Complex Neural Layer

In the lobula-complex neural layer, the T5 neurons integrate signals from the Tm2 and Tm1 neurons in the medulla neural layer. At the same time, the T5 neurons have four sub-types, each of which is sensitive to one of upward, downward, rightward, and leftward moving directions, i.e., have four different direction-selective responses. The calculation formula for the separating operation of T5 is defined in Equation (11):

(11) $$\left\{\begin{array}{l} T5_{U}\left(x,y,t\right)=N_{O\mathrm{FF}}\left(x,y-\mathrm{sd},t\right)\cdot N_{\mathrm{OFF}}^{D}\left(x,y,t\right)-N_{O\mathrm{FF}}\left(x,y,t\right)\cdot N_{\mathrm{OFF}}^{D}\left(x,y-\mathrm{sd},t\right)\\ T5_{D}\left(x,y,t\right)=N_{O\mathrm{FF}}\left(x,y+\mathrm{sd},t\right)\cdot N_{\mathrm{OFF}}^{D}\left(x,y,t\right)-N_{O\mathrm{FF}}\left(x,y,t\right)\cdot N_{\mathrm{OFF}}^{D}\left(x,y+\mathrm{sd},t\right)\\ T5_{R}\left(x,y,t\right)=N_{O\mathrm{FF}}\left(x+\mathrm{sd},y,t\right)\cdot N_{\mathrm{OFF}}^{D}\left(x,y,t\right)-N_{O\mathrm{FF}}\left(x,y,t\right)\cdot N_{\mathrm{OFF}}^{D}\left(x+\mathrm{sd},y,t\right)\\ T5_{L}\left(x,y,t\right)=N_{O\mathrm{FF}}\left(x-\mathrm{sd},y,t\right)\cdot N_{\mathrm{OFF}}^{D}\left(x,y,t\right)-N_{O\mathrm{FF}}\left(x,y,t\right)\cdot N_{\mathrm{OFF}}^{D}\left(x-\mathrm{sd},y,t\right) \end{array}\right.,$$

where $T5_{U}\left(x,y,t\right)$, $T5_{D}\left(x,y,t\right)$, $T5_{R}\left(x,y,t\right)$, and $T5_{L}\left(x,y,t\right)$ represent the signal of upward, downward, rightward, and leftward sub-visual pathways in T5 (see U, D, R, and L of the rectangular dotted box T5 in the lobula-complex neural layer shown in Figure 1); $N_{\mathrm{OFF}}^{D}\left(x,y,t\right)$ represents the delayed normalized OFF signal, which can be obtained by using the temporally delayed output of Tm2 [16,43] (see green TDU and Tm1 in the medulla neural layer shown in Figure 1).

Afterward, the T4 and T5 sub-layers with the same motion direction are integrated into the LPTC in the lobula, namely, the responses of the same motion direction converge to the same sub-layer of the LPTC (see the rectangular dotted box LPTC in the lobula-complex neural layer shown in Figure 1). The calculation formula of the translating moving directions is defined in Equation (12):

(12) $$\left\{\begin{array}{l} LPTC_{U}\left(x,y,t\right)={\left(T4_{U}\left(x,y,t\right)-C_{\mathrm{ON}}\left(x,y,t\right)\right)}^{\gamma_{1}}+{\left(T5_{U}\left(x,y,t\right)-C_{\mathrm{OFF}}\left(x,y,t\right)\right)}^{\gamma_{2}}\\ LPTC_{D}\left(x,y,t\right)={\left(T4_{D}\left(x,y,t\right)-C_{\mathrm{ON}}\left(x,y,t\right)\right)}^{\gamma_{1}}+{\left(T5_{D}\left(x,y,t\right)-C_{\mathrm{OFF}}\left(x,y,t\right)\right)}^{\gamma_{2}}\\ LPTC_{R}\left(x,y,t\right)={\left(T4_{R}\left(x,y,t\right)-C_{\mathrm{ON}}\left(x,y,t\right)\right)}^{\gamma_{1}}+{\left(T5_{R}\left(x,y,t\right)-C_{\mathrm{OFF}}\left(x,y,t\right)\right)}^{\gamma_{2}}\\ LPTC_{L}\left(x,y,t\right)={\left(T4_{L}\left(x,y,t\right)-C_{\mathrm{ON}}\left(x,y,t\right)\right)}^{\gamma_{1}}+{\left(T5_{L}\left(x,y,t\right)-C_{\mathrm{OFF}}\left(x,y,t\right)\right)}^{\gamma_{2}} \end{array}\right.,$$

where $LPTC_{U}\left(x,y,t\right)$, $LPTC_{D}\left(x,y,t\right)$, $LPTC_{R}\left(x,y,t\right)$, and $LPTC_{L}\left(x,y,t\right)$ represent the signal of upward, downward, leftward, and rightward sub-visual pathways of the LPTC; and $\gamma_{1}$ and $\gamma_{2}$ represent the exponents of the ON and OFF signals.

Next, the responses of the opposite motion directions inhibit each other by the LPi. This model adopts the sign-inverting operation to simulate the direction-opponent response (see the rectangular dotted box LPi in the lobula-complex neural layer of Figure 1). The calculation formulas of the vertical and horizontal direction-opponent responses of the LPi are defined in Equations (13) and (14):

(13) $$\left\{\begin{array}{l} \mathrm{VS}\left(t\right)=\sum_{y=1}^{H}\sum_{x=1}^{W}{\left[LPTC_{U}\left(x,y,t\right)-LPi_{U}(x,y,t)\right]}^{+}-\sum_{y=1}^{H}\sum_{x=1}^{W}{\left[LPTC_{D}\left(x,y,t\right)-LPi_{D}(x,y,t)\right]}^{+}\\ \mathrm{HS}\left(t\right)=\sum_{y=1}^{H}\sum_{x=1}^{W}{\left[LPTC_{R}\left(x,y,t\right)-LPi_{R}(x,y,t)\right]}^{+}-\sum_{y=1}^{H}\sum_{x=1}^{W}{\left[LPTC_{L}\left(x,y,t\right)-LPi_{L}(x,y,t)\right]}^{+} \end{array}\right.,$$

(14) $$\left\{\begin{array}{l} LPi_{U}(x,y,t)=LP{\mathrm{TC}}_{D}(x,y,t)\\ LPi_{D}(x,y,t)=LP{\mathrm{TC}}_{U}(x,y,t)\\ LPi_{L}(x,y,t)=LP{\mathrm{TC}}_{R}(x,y,t)\\ LPi_{R}(x,y,t)=LP{\mathrm{TC}}_{L}(x,y,t) \end{array}\right.,$$

where $\mathrm{VS}\left(t\right)$ and $\mathrm{HS}\left(t\right)$ represent the direction-opponent responses of vertical and horizontal; H and W represent height and width of the two-dimensional visual field in the retina neural layer; $\mathrm{VS}\left(t\right)>0$ represents the upward motion and $\mathrm{VS}\left(t\right)<0$ represents the downward motion; and $\mathrm{HS}\left(t\right)>0$ represents the rightward motion and $\mathrm{HS}\left(t\right)<0$ represents the leftward motion (see MDTO in the lobula-complex neural layer shown in Figure 1).

The pseudo-code of the proposed bio-inspired model is depicted in Algorithm 1, which shows the signal processing of the proposed bio-inspired model in detail.
**Algorithm 1.** Proposed bio-inspired model1: Input: Visual stimulus sequences at continuous frames, $L\left(x,y,t\right)<t=1,2,3,\ldots ,n-2,n-1,n>$. 2: Output: Motion directions of translating objects, $\mathrm{VS}\left(t\right)$ and $\mathrm{HS}\left(t\right)$. 3: Procedure ProposedBioInspiredModel _CALCULATION($L_{t-1}$, $L_{t}$) 4:   for num = from ($L_{1}$, $L_{2}$) to ($L_{n-1}$, $L_{n}$) 5:     // 1. Retina neural layer 6:     Compute the gray-scale successive frames and the change in luminance between every two successive frames 7:    // 2. Lamina neural layer 8:     Firstly, compute the ON and OFF signals by the HWR algorithm, get $L_{\mathrm{ON}/\mathrm{OFF}}\left(x,y,t\right)$ 9:     Finally, compute the pre-processed ON and OFF signals by the early visual pre-processing in Equations (1)–(4), the threshold comparison, and LI, get $S_{\mathrm{ON}/\mathrm{OFF}}\left(x,y,t\right)$ 10:    // 3. Medulla neural layer 11:    Firstly, compute the normalized ON and OFF signals by Equations (5)–(7), get $N_{\mathrm{ON}/\mathrm{OFF}}\left(x,y,t\right)$ 12:    Secondly, compute the local contrast ON and OFF pathway signals by Equations (8) and (9), get $C_{\mathrm{ON}/\mathrm{OFF}}\left(x,y,t\right)$ 13:    Finally, compute the sub-visual signals of four different motion directions of the T4 by Equation (10), get $T4_{X\in \left\{U,D,R,L\right\}}\left(x,y,t\right)$       // 4. Lobula-complex neural layer 14:    Firstly, compute the sub-visual signals of four different motion directions of the T5 by Equation (11), get $T5_{X\in \left\{U,D,R,L\right\}}\left(x,y,t\right)$ 15:    Secondly, compute the integrated sub-visual motion and contrast pathway signals of the LPTC by Equation (12), get $LPTC_{X\in \left\{U,D,R,L\right\}}\left(x,y,t\right)$ 16:    Finally, compute the vertical and horizontal direction-opponent responses of the LPi by Equations (13) and (14), get $\mathrm{VS}\left(t\right)$ and $\mathrm{HS}\left(t\right)$ 17:    return $\mathrm{VS}\left(t\right)$ and $\mathrm{HS}\left(t\right)$ 18:   end for 19: End procedure

### 3.5. Model Parameter Configuration

The parameter configuration of the proposed bio-inspired model is given in Table 4. All of the parameters are determined empirically by considering the functionality of the visual neural system of Drosophila.

## 4. Results

In the experiments, the proposed bio-inspired model was set up in Visual Studio 2010 (The Microsoft Corporation, Redmond, WA, USA) using Intel Core i7-8700 and 3.20 GHz (12 CPUs) hardware. The synthetic visual stimulus sequences were generated by Vision Egg [49], and the noise was added to the synthetic visual stimulus sequences by OpenCV 2.4.9 (The Intel Corporation, Santa Clara, CA, USA). The performance of the proposed bio-inspired model was verified by the ablation study, the detection performance of translating objects in pure and real-world complex noise backgrounds, and further investigations.

### 4.1. Ablation Study

**Figure 2:**
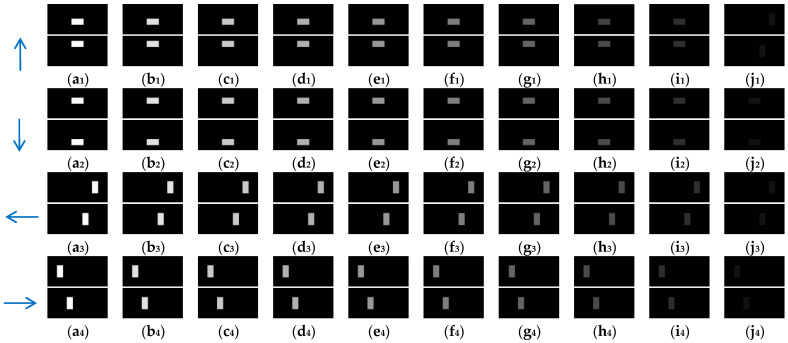
Sample frames 40 and 80 from 10 groups of synthetic pure visual stimulus sequences. The light blue arrows represent the motion direction of the translating object. (**a_1_**–**j_1_**) represent the translating object in the upward motion; (**a_2_**–**j_2_**) represent the translating object in the downward motion; (**a_3_**–**j_3_**) represent the translating object in the leftward motion; (**a_4_**–**j_4_**) represent the translating object in the rightward motion. The gray values of the translating objects in $a_{m}$, $b_{m}$, $c_{m}$, $d_{m}$, $e_{m}$, $f_{m}$, $g_{m}$, $h_{m}$, $i_{m}$, and $j_{m}$$\left\{m\in \left(1,2,3,4\right)\right\}$ are 250, 225, 200, 175, 150, 125, 100, 75, 50, and 25, respectively.

**Figure 3:**
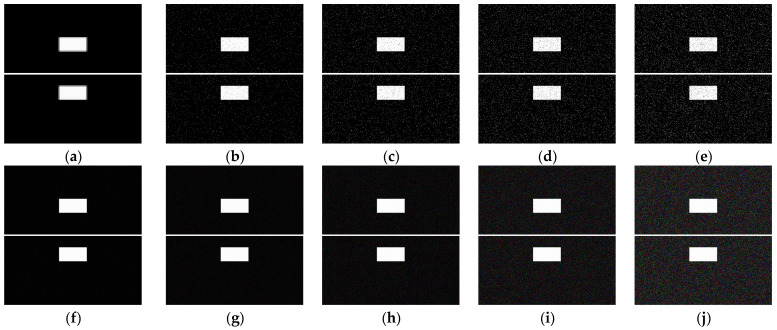
Sample frames 40 and 80 from 10 groups of the synthetic pure noisy visual stimulus sequences with the upward moving object based on dataset II. The noise intensity in (**a**), (**b**), (**c**), (**d**), (**e**), (**f**), (**g**), (**h**), (**i**) and (**j**) are 0, SPN = 0.01, SPN = 0.02, SPN = 0.03, SPN = 0.04, GN = 10.0, GN = 20.0, GN = 30.0, GN = 50.0, and GN = 80.0, respectively.

In this section, 10 groups of the synthetic pure visual stimulus sequences and 10 groups of the synthetic pure noisy visual stimulus sequences were adopted, the pure background is a solid image with a fixed gray value of 1, and the resolution of the pure background is 500 × 250 pixels × pixels. The translating object is a solid rectangle with a different gray value that varies between a maximum of 250 and a minimum of 25, and is taken every 25 gray values; the motion velocity of the translating object is 2000 pixels/second. In the upward/downward motion, the size of the translating object is 100 × 50 pixels × pixels. In the leftward/rightward motion, the size of the translating object is 50 × 100 pixels × pixels. Each synthetic pure visual stimulus sequence consists of 100 frames. Sample frames 40 and 80 from the 10 groups of the synthetic pure visual stimulus sequences are shown as dataset I in Figure 2. We adopted dataset I (no noise, only various contrasts in the figure-ground) to verify the effectiveness of the contrast neural computation. For dataset II, salt and pepper noise (SPN ratio: 0.01/0.02/0.03/0.04) or Gaussian noise (GN standard deviation: 10.0/20.0/30.0/50.0/80.0) were added based on Figure 2(a_1_–a_4_); sample frames 40 and 80 from the 10 groups of the synthetic pure noisy visual stimulus sequences with the upward moving object are shown in Figure 3; in the same way, the synthetic pure noisy visual stimulus sequences with the downward, leftward, and rightward moving object were obtained. We adopted dataset II (keep the figure-ground contrast constant, only various noises) to verify the effectiveness of the spatial denoising mechanism.

**Figure 4:**
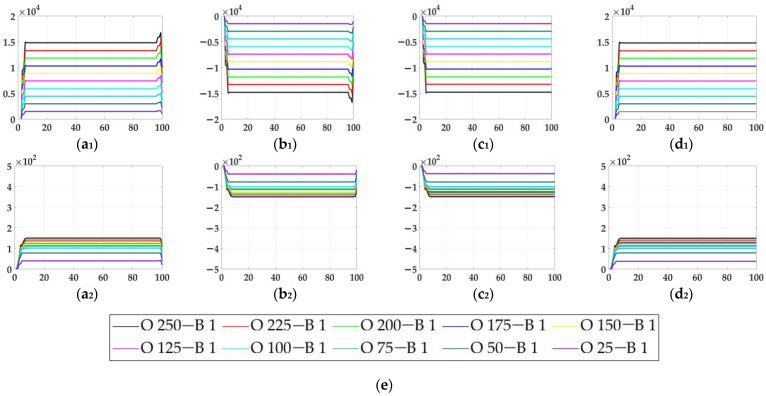
The experimental results of model responses based on dataset I. Horizontal axis represents time in frames; vertical axis represents model responses. (**a_1_**,**a_2_**) represent the translating object in the upward motion; (**b_1_**,**b_2_**) represent the translating object in the downward motion; (**c_1_**,**c_2_**) represent the translating object in the leftward motion; (**d_1_**,**d_2_**) represent the translating object in the rightward motion. (**a_1_**–**d_1_**) represent the model responses of Model 1 and (**a_2_**–**d_2_**) represent the model responses of the proposed bio-inspired model; (**e**) represents the legend for (**a_1_**–**d_2_**). O represents the translating object and B represents the background.

**Figure 5:**
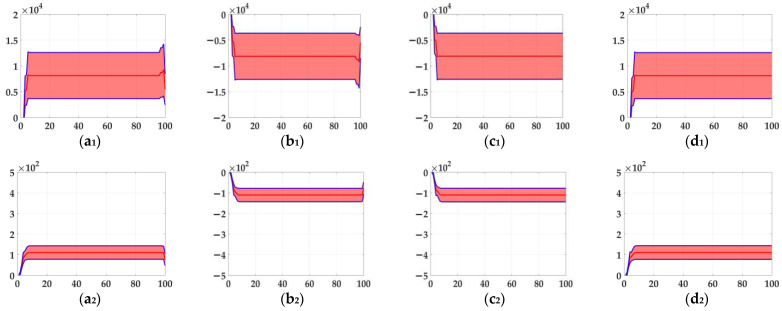
The experimental results of the mean and standard deviation based on dataset I. Horizontal axis represents time in frames; vertical axis represents the mean and standard deviation. (**a_1_**,**a_2_**) represent the translating object in the upward motion; (**b_1_**,**b_2_**) represent the translating object in the downward motion; (**c_1_**,**c_2_**) represent the translating object in the leftward motion; (**d_1_**,**d_2_**) represent the translating object in the rightward motion; (**a_1_**–**d_1_**) represent the mean and standard deviation of Model 1; and (**a_2_**–**d_2_**) represent the mean and standard deviation of the proposed bio-inspired model.

**Figure 6:**
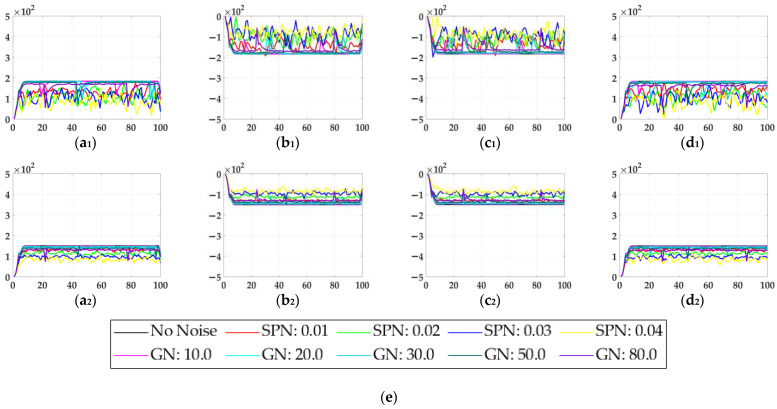
The experimental results of model responses based on dataset II. Horizontal axis represents time in frames; vertical axis represents model responses. (**a_1_**,**a_2_**) represent the translating object in the upward motion; (**b_1_**,**b_2_**) represent the translating object in the downward motion; (**c_1_**,**c_2_**) represent the translating object in the leftward motion; (**d_1_**,**d_2_**) represent the translating object in the rightward motion; (**a_1_**–**d_1_**) represent the model responses of Model 2; and (**a_2_**–**d_2_**) represent the model responses of the proposed bio-inspired model. (**e**) represents the legend of for (**a_1_**–**d_2_**). SPN represents the salt and pepper noise; GN represents the Gaussian noise.

**Figure 7:**
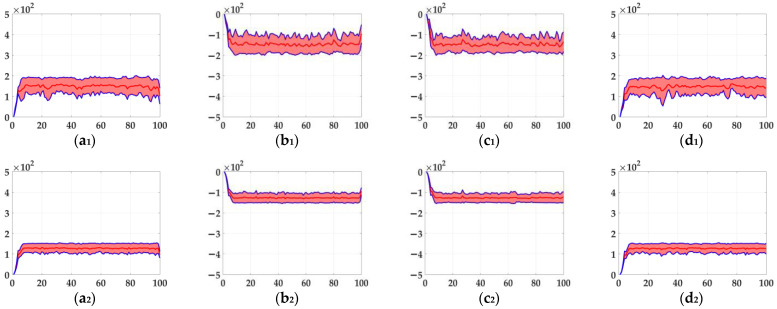
The experimental results of the mean and standard deviation based on dataset II. Horizontal axis represents time in frames; vertical axis represents the mean and standard deviation. (**a_1_**,**a_2_**) represent the translating object in the upward motion; (**b_1_**,**b_2_**) represent the translating object in the downward motion; (**c_1_**,**c_2_**) represent the translating object in the leftward motion; (**d_1_**,**d_2_**) represent the translating object in the rightward motion; (**a_1_**–**d_1_**) represent the mean and standard deviation of Model 2; and (**a_2_**–**d_2_**) represent the mean and standard deviation of the proposed bio-inspired model.

In the ablation study, the proposed bio-inspired model without the ON and OFF contrast neural computation (i.e., Model 1) and the proposed bio-inspired model without the spatial denoising mechanism (i.e., Model 2) were adopted to verify the effectiveness of the proposed bio-inspired model. The experimental results of model responses based on dataset I are shown in Figure 4. The experimental results of the mean and standard deviation based on dataset I are shown in Figure 5. The experimental results of model responses based on dataset II are shown in Figure 6. The experimental results of the mean and standard deviation based on dataset II are shown in Figure 7.

Since the standard deviation is affected by the value of the variable itself, we adopted the dispersion of the coefficient of variation to further compare. The coefficient of variation, the inter-quartile range (IQR) of the coefficient of variation, and the sum of the coefficient of variation are defined in Equations (15)–(17):

(15) $$V_{c}\left(t\right)=\left|\frac{\sigma \left(t\right)}{m\left(t\right)}\right|,$$

(16) $$\mathrm{IQR}=Q_{3}-Q_{1},$$

(17) $$S=\sum_{t}V_{c}\left(t\right),$$

where $V_{c}\left(t\right)$ represents the coefficient of variation, $\sigma \left(t\right)$ represents the standard deviation, $m\left(t\right)$ represents the mean value, t represents the temporal coordinate, IQR represents the inter-quartile range of the coefficient of variation, $Q_{3}$ represents the upper quartile, $Q_{1}$ represents the lower quartile, and S represents the sum of coefficient of variation. The smaller the inter-quartile range, the better the statistical result; the smaller the coefficient of variation and the sum of the coefficient of variation, the better the statistical result. The experimental results of the coefficient of variation and the sum of the coefficient of variation based on dataset I comparison in Model 1 and the proposed bio-inspired model are shown in Table 5. The experimental results of the coefficient of variation and the sum of the coefficient of variation based on dataset II comparison in Model 2 and the proposed bio-inspired model are shown in Table 6.

From Figure 4 and Figure 5, we can draw the following two conclusions. (1) The proposed bio-inspired model and Model 1 have a high response to the translating object in the upward, downward, leftward, and rightward motions; furthermore, they can accurately detect the motion direction of the translating object. (2) In the upward/downward motion of the translating object, the horizontal response is approximately 0; furthermore, in the leftward/rightward motion of the translating object, the vertical response is approximately 0. From Table 5, compared with Model 1, we can draw the following two conclusions. (1) The inter-quartile range of the proposed bio-inspired model is reduced by 30.03%. (2) The sum of the coefficient of variation in the proposed bio-inspired model is reduced by 42.48%. In summary, the contrast neural computation of the proposed bio-inspired model effectively reduces the fluctuation in the model response under various contrasts in the figure-ground.

From Figure 6 and Figure 7, we can draw the following two conclusions. (1) The proposed bio-inspired model and Model 2 have a strong response to the translating object in the upward, downward, leftward, and rightward motions, and can accurately detect the motion direction of the translating object; furthermore, the fluctuation in the proposed bio-inspired model is significantly lower than that in Model 2. (2) In the upward/downward motion of the translating object, the horizontal response is small relative to the vertical response; furthermore, in the leftward/rightward motion of the translating object, the vertical response is small relative to the horizontal response. From Table 6, compared with Model 2, we can draw the following two conclusions. (1) The inter-quartile range of the coefficient of variation in the proposed bio-inspired model is reduced by 57.38%. (2) The sum of the coefficient of variation in the proposed bio-inspired model is reduced by 17.36%. In summary, the spatial denoising mechanism of the proposed bio-inspired model has the ability to suppress environmental noise and effectively reduces the fluctuation in the model response under different noise types with different intensities.

### 4.2. Detection Performance of Motion Directions of Translating Objects in Pure Noise Backgrounds

**Figure 8:**
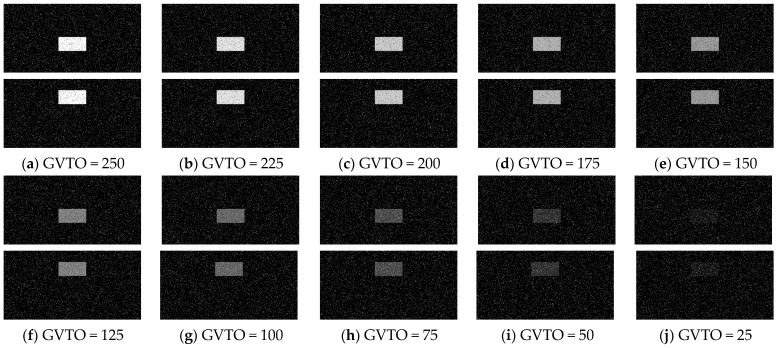
Sample frames 40 and 80 from 10 groups of the synthetic pure noising visual stimulus sequences with the upward moving object based on dataset III (SPN ratio: 0.04). The gray values of the translating objects (GVTO) in (**a**), (**b**), (**c**), (**d**), (**e**), (**f**), (**g**), (**h**), (**i**) and (**j**) are 250, 225, 200, 175, 150, 125, 100, 75, 50, and 25, respectively.

**Figure 9:**
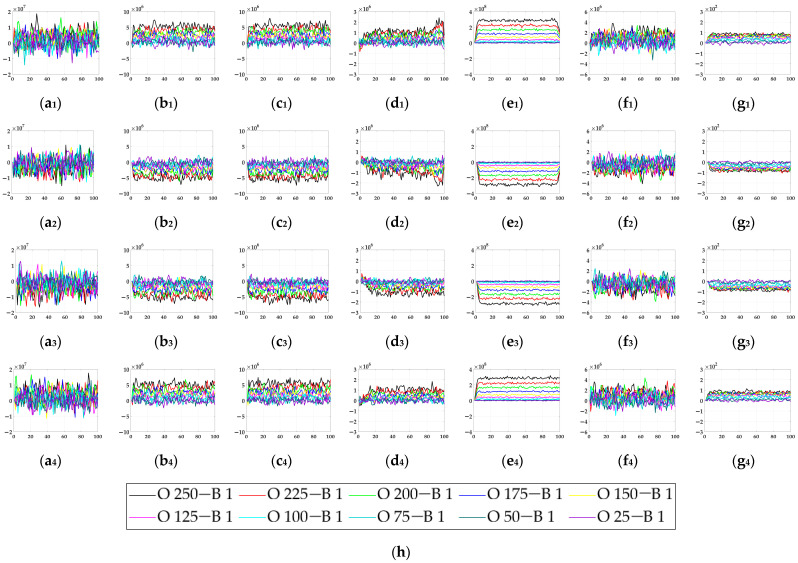
The experimental results of model responses based on dataset III (SPN ratio: 0.04). Horizontal axis represents time in frames; vertical axis represents model responses. (**a_1_**–**g_1_**) represent the translating object in the upward motion; (**a_2_**–**g_2_**) represent the translating object in the downward motion; (**a_3_**–**g_3_**) represent the translating object in the leftward motion; (**a_4_**–**g_4_**) represent the translating object in the rightward motion. $a_{m}$, $b_{m}$, $c_{m}$, $d_{m}$, $e_{m}$, $f_{m}$, and $g_{m}$$\left\{m\in \left(1,2,3,4\right)\right\}$ represent EMD [9], TQD [12], FQD [13], FU [16], XU [38], WANG [50], and the proposed bio-inspired model, respectively; (**h**) represents the legend of (**a_1_**–**g_4_**). O represents the translating object and B represents the background.

**Figure 10:**
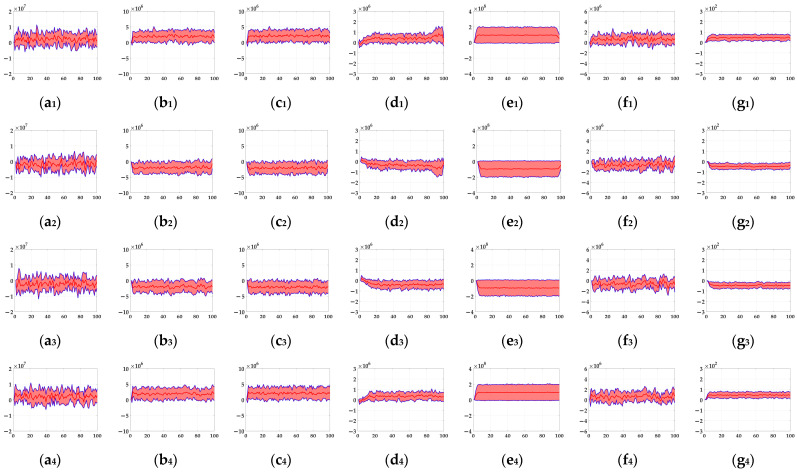
The experimental results of the mean and standard deviation based on dataset III (SPN ratio: 0.04). Horizontal axis represents time in frames; vertical axis represents the mean and standard deviation. (**a_1_**–**g_1_**) represent the translating object in the upward motion; (**a_2_**–**g_2_**) represent the translating object in the downward motion; (**a_3_**–**g_3_**) represent the translating object in the leftward motion; (**a_4_**–**g_4_**) represent the translating object in the rightward motion. $a_{m}$, $b_{m}$, $c_{m}$, $d_{m}$, $e_{m}$, $f_{m}$, and $g_{m}$$\left\{m\in \left(1,2,3,4\right)\right\}$ represent EMD [9], TQD [12], FQD [13], FU [16], XU [38], WANG [50], and the proposed bio-inspired model, respectively.

In this section, six kinds of comparable bio-inspired models (i.e., SotA models) including the EMD [9], TQD [12], FQD [13], FU [16], XU [38], and WANG [50] models were adopted to verify the detection performance of the proposed bio-inspired model; the value of the sampling distance (sd) of the EMD, TQD, and FQD models is 4, and the value of the parameter of the FU and XU models is shown in the references [16] and [38], and remains unchanged. To maintain consistency with the structure of other models, the WANG model adopts a combination of GPS, Gaussian blur, difference calculation, HWR, LI, and TQD, and the value of the sd is 4. SPN ratio (0.01/0.02/0.03/0.04) or GN standard deviation (10.0/20.0/30.0/50.0/80.0) were added based on Figure 2, as dataset III, i.e., sample frames 40 and 80 from 10 groups of the synthetic pure noisy visual stimulus sequences (SPN ratio: 0.04) with the upward moving object that is shown in Figure 8. The experimental results of model responses based on dataset III (SPN ratio: 0.04) are shown in Figure 9. The experimental results of the mean and standard deviation based on dataset III (SPN ratio: 0.04) are shown in Figure 10. The experimental results of the detection success rate based on the dataset III comparison for the six kinds of bio-inspired models and the proposed bio-inspired model are shown in Table 7. The experimental results of the inter-quartile range of the coefficient of variation based on dataset III comparison for the six kinds of bio-inspired models and the proposed bio-inspired model are shown in Table 8. The experimental results of the inter-quartile range and the sum of the coefficient of variation based on dataset III comparison for the six kinds of bio-inspired models and the proposed bio-inspired model are shown in Table 9.

To compare the detection performance of the bio-inspired models, we adopt the detection success rate for quantitative analysis, which is defined in Equation (18):

(18) $$D_{R}=\frac{\mathrm{number}\;\mathrm{of}\;\mathrm{true}\;\mathrm{detections}}{\mathrm{number}\;\mathrm{of}\;\mathrm{frame}\;\mathrm{sequences}}$$

where $D_{R}$ represents the detection success rate. The experimental results of the detection success rate $D_{R}$ comparison for the six kinds of bio-inspired models and the proposed bio-inspired model are shown in Table 7.

From Figure 9 and Figure 10 and Table 7, we can draw the following three conclusions. (1) The EMD [9], TQD [12], FQD [13], FU [16], XU [38], WANG [50], and proposed bio-inspired models have strong responses to the translating object in the upward, downward, leftward, and rightward motions; however, they have some detection errors of varying degrees. (2) The detection performance of the proposed bio-inspired model is superior to the six kinds of comparable bio-inspired models in the detection success rate; surprisingly, in the ten sub-datasets of dataset III, eight sub-datasets achieve the first best results, one sub-dataset achieves the second best result, and one sub-dataset achieves the third best result. Specifically, the detection success rate of the proposed bio-inspired model is increased by 9.32%, 3.59%, 2.72%, 5.84%, 6.27%, and 4.51%, respectively. The average detection success rate of the proposed bio-inspired model is increased by 5.38%. Thus, the proposed bio-inspired model can robustly detect the motion direction of the translating object. (3) In the upward/downward motion of the translating object, the horizontal response is small relative to the vertical response; furthermore, in the leftward/rightward motion of the translating object, the vertical response is small relative to the horizontal response. From Table 8 and Table 9, comparing the six kinds of bio-inspired models, we can draw the following two conclusions. (1) In ten groups of experiments, the inter-quartile range of the coefficient of variation in the proposed bio-inspired model achieves three of the first results and four of the second results. The inter-quartile range of the coefficient of variation in the proposed bio-inspired model is reduced by 94.70%, 65.21%, 57.85%, 74.32%, -150.64%, and 91.16%, respectively. The average decline in the inter-quartile range of the coefficient of variation in the proposed bio-inspired model is 38.77%. (2) In 10 groups of experiments, the sum of the coefficient of variation in the proposed bio-inspired model achieves ten of the first results. The sum of the coefficient of variation in the proposed bio-inspired model is reduced by 66.41%, 51.01%, 48.88%, 55.89%, 60.82%, and 59.17%, respectively. The average decline in the sum of the coefficient of variation in the proposed bio-inspired model is 57.03%. To summarize, the proposed bio-inspired model can robustly detect the motion direction of the translating object and effectively reduces the fluctuation in the model response against variable contrast in the figure-ground and environmental noise interference, which verifies the most basic detection capabilities of the proposed bio-inspired model.

### 4.3. Detection Performance of Motion Directions of Translating Objects in Real-World Complex Noise Backgrounds

**Figure 11:**
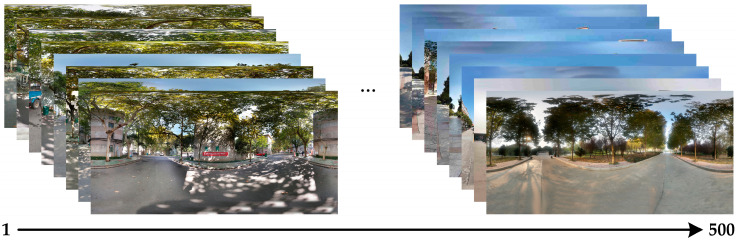
The sample of panoramic real-world complex backgrounds (ID: 1–500) amongst dataset IV, including 500 panoramic real-world backgrounds used in the tests. … represents other panoramic real-world complex backgrounds.

**Figure 12:**
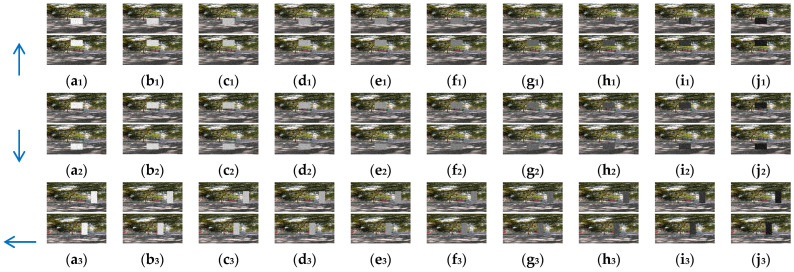

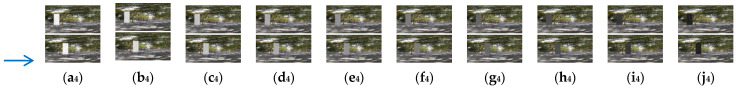
Sample frames 40 and 80 from 10 groups of synthetic real-world complex noisy visual stimulus sequences from one sample (ID = 8) of dataset IV (SPN ratio: 0.04). The light blue arrows represent the motion direction of the translating object. (**a_1_**–**j_1_**) represent the translating object in the upward motion; (**a_2_**–**j_2_**) represent the translating object in the downward motion; (**a_3_**–**j_3_**) represent the translating object in the leftward motion; and (**a_4_**–**j_4_**) represent the translating object in the rightward motion. The gray values of the translating objects in $a_{m}$, $b_{m}$, $c_{m}$, $d_{m}$, $e_{m}$, $f_{m}$, $g_{m}$, $h_{m}$, $i_{m}$, and $j_{m}$$\left\{m\in \left(1,2,3,4\right)\right\}$ are 250, 225, 200, 175, 150, 125, 100, 75, 50, and 25, respectively.

**Figure 13:**
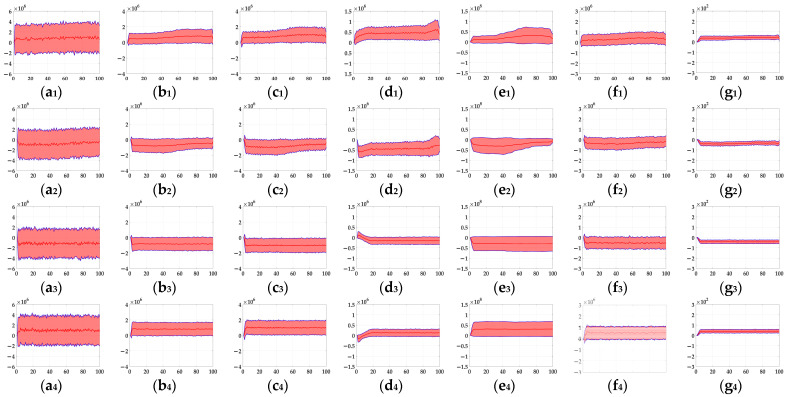
The experimental results of the mean and standard deviation based on dataset IV (SPN ratio: 0.04). Horizontal axis represents time in frames; vertical axis represents the mean and the standard deviation. The red line represents the mean, the transparent red shadow represents the standard deviation, and the blue lines represent the addition and subtraction between the mean and the standard deviation; (**a_1_**–**g_1_**) represent the translating object in the upward motion; (**a_2_**–**g_2_**) represent the translating object in the downward motion; (**a_3_**–**g_3_**) represent the translating object in the leftward motion; (**a_4_**–**g_4_**) represent the translating object in the rightward motion.$a_{m}$, $b_{m}$, $c_{m}$, $d_{m}$, $e_{m}$, $f_{m}$, and $g_{m}$$\left\{m\in \left(1,2,3,4\right)\right\}$ represent EMD [9], TQD [12], FQD [13], FU [16], XU [38], WANG [50], and the proposed bio-inspired model, respectively.

**Figure 14:**
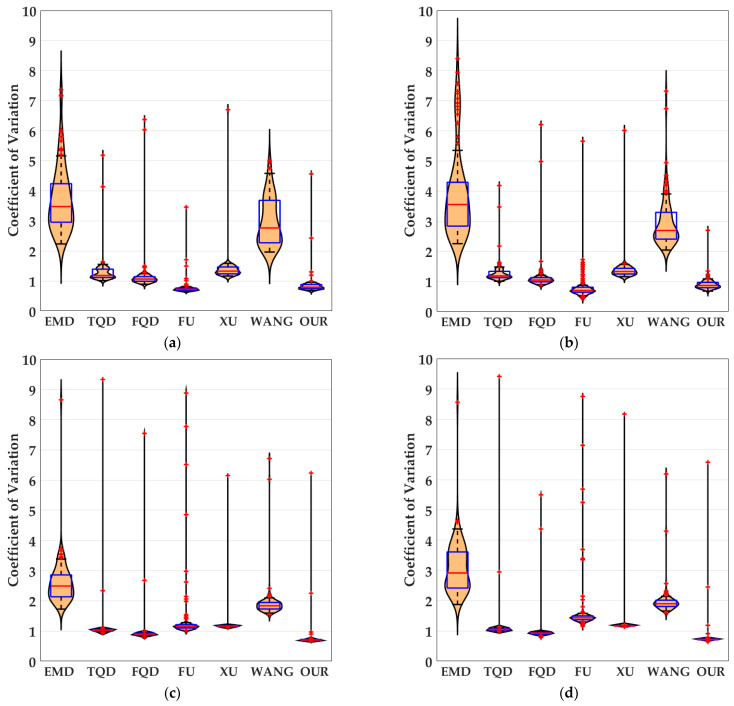
The experimental results shown as violin- and box-type diagrams of the coefficient of variation based on dataset IV (SPN ratio: 0.04), comparison drawings for the 6 kinds of bio-inspired models and the proposed bio-inspired model: (**a**) represents the translating object in the upward motion; (**b**) represents the translating object in the downward motion; (**c**) represents the translating object in the leftward motion; (**d**) represents the translating object in the rightward motion. OUR represents the proposed bio-inspired model [9,12,13,16,38,50].

In this section, to further verify the detection performance of the proposed bio-inspired model in real-world complex backgrounds, we used a Samsung camera (8 megapixels) to collect 500 real-world scenes on campus and the institute, including trees, buildings, bicycles, and so on. Each real-world scene of the campus and the institute comprised 60 partially overlapping images that were stitched together with PTGui (New House Internet Services BV, Rotterdam, Netherlands) to obtain the panoramic real-world images [51]; the resolution of the panoramic real-world images is set to 2048 × 310 pixels × pixels. These panoramic real-world images are the backgrounds, and the translating object is embedded in the panoramic real-world background. The translating object is a solid rectangle with a different gray value that varies between a maximum of 250 and a minimum of 25, and is taken every 25 gray values. In the upward/downward motion, the size and the motion velocity of the translating object are 100 × 50 pixels × pixels and 2000 pixels/second. In the leftward/rightward motion, the size and the motion velocity of the translating object are 50 × 100 pixels × pixels and 2000 pixels/second. Each panoramic background consists of 40 groups of real-world visual stimulus sequences and each group of the real-world visual stimulus sequences consists of 100 frames, the resolution and frame rate of each group of the real visual stimulus sequences are 500 × 250 pixels × pixels and 30 fps. The motion velocities of the background in the leftward/rightward and upward/downward motion are 500 and 2000 pixels/second, respectively. The sample of panoramic real-world backgrounds (ID: 1–500) amongst dataset including 500 testing panoramic real-world complex backgrounds are shown in Figure 11. The SPN ratio (0.01/0.02/0.03/0.04) or GN standard deviation (10.0/20.0/30.0/50.0/80.0) were added based on Figure 11, as dataset IV; sample frames 40 and 80 from 10 groups of synthetic real-world complex noisy visual stimulus sequences from one sample (ID = 8) of dataset IV (SPN ratio: 0.04) are shown in Figure 12. The experimental results of the mean and standard deviation based on dataset IV (SPN ratio: 0.04) are shown in Figure 13. The experimental results, represented by violin- and box-type diagrams of the coefficient of variation based on dataset IV (SPN ratio: 0.04), comparison drawings for the six kinds of bio-inspired models, and the proposed bio-inspired model, are shown in Figure 14. The denser the distribution of the coefficient of variation is, the better the statistical result. The closer the coefficient of variation is to 0, the better the statistical result. Therefore, we hope that the height of the violin based on the coefficient of variation can be as narrow as possible, and the position of the violin based on the coefficient of variation can be as close to 0 as possible. In addition, the blue box in each violin represents a box-plot, which is used to characterize the dispersion degree of the coefficient of variation; the blue top and bottom of each box-plot represent the upper and lower quartile, the red line of each box-plot represents the median value, the black top and bottom line of each box-plot represent the upper and lower edges, and the red crosses represent outliers. The experimental results of the detection success rate based on the dataset IV comparison for the six kinds of bio-inspired models and the proposed bio-inspired model are shown in Table 10. The experimental results of the inter-quartile range of the coefficient of variation based on dataset IV comparison for the six kinds of bio-inspired models and the proposed bio-inspired model are shown in Table 11. The experimental results of the sum of the coefficient of variation based on dataset IV comparison for the six kinds of bio-inspired models and the proposed bio-inspired model are shown in Table 12.

From Figure 13 and Table 10, we can draw the following four conclusions. (1) From Figure 11(a_1_–a_4_), the detection performance of the EMD [9] is not satisfactory. (2) From Figure 11(b_1_–f_1_,b_2_–f_2_,b_3_–f_3_,b_4_–f_4_), the detection performance of the TQD [12], FQD [13], FU [16], XU [38], and WANG [50] models can meet the basic detection performance, but there are still a few detection errors. (3) The detection performance of the proposed bio-inspired model is superior to the six kinds of comparable bio-inspired models in the detection success rate; surprisingly, in the ten sub-datasets of dataset IV, seven sub-datasets achieve the first best results and two sub-datasets achieve the second best results. Specifically, the detection success rate of the proposed bio-inspired model is increased by 10.82%, 3.33%, 3.15%, 4.16%, 6.05%, and 4.26%, respectively. The average increase in the detection success rate of the proposed bio-inspired model is 5.30%. So, the proposed bio-inspired model can robustly detect the motion direction of the translating object. (4) In the upward/downward motion of the translating object, the horizontal response is small relative to the vertical response; furthermore, in the leftward/rightward motion of the translating object, the vertical response is small relative to the horizontal response. From Figure 14, we can draw the following two conclusions. (1) In the upward motion, the height of the proposed bio-inspired model violin diagram is narrower than the EMD [9], TQD [12], FQD [13], XU [38], and WANG [50] models, but not that of the FU [16] model. In the downward motion, the height of the proposed bio-inspired model violin diagram is narrower than the EMD [9], TQD [12], XU [38], and WANG [50] models, but not that of the FQD [13] and FU [16] models. In the leftward motion, the height of the proposed bio-inspired model violin diagram is narrower than the six kinds of comparable bio-inspired models. In the rightward motion, the height of the proposed bio-inspired model violin diagram is narrower than the EMD [9], TQD [12], FQD [13], FU [16], and WANG [50] models, but not that of the XU [38] model. (2) In the upward/downward motion, the position of the violin diagram for the proposed bio-inspired model is closer to 0 than the EMD [9], TQD [12], FQD [13], XU [38], and WANG [50] models, but not closer than that for the FU [16] model. In the leftward/rightward motion, the position of the violin diagram for the proposed bio-inspired model is closer to 0 than for the six kinds of comparable bio-inspired models. From Table 11 and Table 12, we can draw the following two conclusions. (1) The inter-quartile range of the coefficient of variation in the proposed bio-inspired model is reduced by 84.83%, 10.02%, 23.23%, 27.52%, 64.32%, and 77.12%. The average decline in the inter-quartile range of the coefficient of variation in the proposed bio-inspired model is 47.84%. (2) The sum of the coefficient of variation in the proposed bio-inspired model is reduced by 71.49%, 66.47%, 64.78%, 64.45%, 69.49%, and 68.16%. The average decline in the sum of the coefficient of variation in the proposed bio-inspired model is 67.47%. Briefly, the proposed bio-inspired model can robustly detect the motion direction of the translating object and effectively reduces the fluctuation in the model response under various contrasts in the figure-ground of real-world complex noise backgrounds.

### 4.4. Further Investigations

To further estimate the performance of the proposed bio-inspired model, the spatial denoising mechanism is replaced by the difference of Gaussians (DoG) [22] (Model 3), fast-depolarizing slow-repolarizing (FDSR) [52] (Model 4), Gaussian filter (Model 5), mean filter (Model 6), or median filter (Model 7) based on two sub-datasets (SPN ratio: 0.04 and GN standard deviation: 80.0) of dataset IV, for further comparison. The DoG and FDSR as early visual pre-processing mechanisms have previously been validated for their role in filtering background interference [22,52]. Gaussian, mean, and median filters are standard engineering noise-filtering methods [53].

From Table 13, comparing Models 3, 4, 5, 6, and 7, we can draw the following two conclusions. (1) The inter-quartile range of the coefficient of variation in the proposed bio-inspired model is reduced by 76.26%, 34.46%, 28.32%, 25.86%, and −1.7%, respectively. The average decline in the inter-quartile range of the coefficient of variation in the proposed bio-inspired model is 32.64%. (2) The sum of the coefficient of variation in the proposed bio-inspired model is reduced by 47.64%, 25.48%, 33.69%, 26.96%, and 1.3%, respectively. The average decline in the sum of the coefficient of variation in the proposed bio-inspired model is 27.01%. From Table 14, comparing Models 3, 4, 5, 6, and 7, we can draw the following two conclusions. (1) The inter-quartile range of the coefficient of variation in the proposed bio-inspired model is reduced by 7.87%, −0.82%, −2.50%, 13.99%, and 4.65%, respectively. The average decline in the inter-quartile range of the coefficient of variation in the proposed bio-inspired model is 4.64%. (2) The sum of the coefficient of variation in the proposed bio-inspired model is reduced by −18.65%, −26.94%, −18.22%, 2.30%, and 1.57%, respectively. The average decline in the sum of the coefficient of variation in the proposed bio-inspired model is -11.99%. To summarize, for the salt and pepper noise (SPN ratio: 0.04), the performance of the proposed bio-inspired model is superior to that of Models 3, 4, 5, and 6, and is close to that of Model 7. For the Gaussian noise (GN standard deviation: 80.0), the performance of the proposed bio-inspired model obtains the third best result according to the inter-quartile range of the coefficient of variation; the performance of the proposed bio-inspired model is superior to Models 6 and 7 but inferior to Models 3, 4, and 5, according to the sum of the coefficient of variation.

## 5. Discussion

In the lamina neural layer, the spatial denoising mechanism is regarded as the early visual pre-processing, which can suppress environmental noise of the ON and OFF visual pathways. In the medulla neural layer, the non-linear instantaneous feedback divisive contrast normalization mechanism dynamically suppresses the neural signals to reduce local contrast sensitivity, and the parallel contrast pathways are activated. In the lobula-complex neural layer, the parallel motion and contrast pathways converge and the contrast pathways negatively affect the motion pathways to suppress the high-contrast optical flow. The proposed bio-inspired model performs feed-forward visual processing in a four-hierarchical neural network to denoise and encode polar motion and contrast information, respectively. The contrast neural computation and the spatial denoising mechanism are the main innovations of this modeling study because they act as an instantaneous, feedback, and dynamic normalization mechanism and environmental noise suppression for processing ON and OFF signals. There are ON and OFF contrast pathways to neutralize high contrast local ON and OFF motion-induced excitation for robust and stable model responses against variable contrast in the figure-ground of the pure and real-world complex noise backgrounds. To confirm the proposed bio-inspired model, we produced four datasets consisting of the translating motions of a rectangle object against the pure and real-world complex backgrounds with different noise types with variable intensities. To highlight the detection performance of the proposed bio-inspired model, comparative experiments were carried out. Comparative experiments consist of four sections. Firstly, the ablation study verifies the effectiveness of the contrast neural computation and the spatial denoising mechanism. Secondly, the pure background verifies the effectiveness of the proposed bio-inspired model under different noise types with variable intensities and different gray-scale objects against the same gray-scale background. Thirdly, the real-world complex background verifies the effectiveness of the proposed bio-inspired model under different noise types with variable intensities and different gray-scale objects against different real-world complex backgrounds. Finally, further investigations verify the performance of the proposed bio-inspired model under various contrasts in the figure-ground of real-world complex noise backgrounds. The experimental results verify the proposed bio-inspired model has better stability and robustness for detection in pure and real-world complex backgrounds with different noise types. Separating motion and contrast into ON and OFF pathways, the model works effectively to alleviate the response fluctuation against variable contrast in the figure-ground; furthermore, the spatial denoising mechanism makes the bio-inspired visual neural model more robust and stable in response to translating motion. Future research efforts may involve the following aspects:

(1) The limitation of this study is that the baseline contrast sensitivity has an impact on the detection performance of the proposed bio-inspired model. Therefore, how to effectively determine the corresponding relationship between variable signals and the baseline contrast sensitivity is the focus of future research.
(2) How to use the moving direction of the translating object based on LPTC neurons to detect the wide-field or local-salient object is one of the future research points.
(3) Integrating the probability between layers of the LPTC-based bio-inspired model to further improve environmental interference is one of the future research points.

## 6. Conclusions

In this study, the LPTC is used as a research object to study its physiological mechanism and the existing computational model. We find that it can detect the motion direction of the translating object robustly and stably under a complex dynamic noise background; based on this biological vision paradigm, a bio-inspired visual neural model is proposed, which incorporates continuous computational neural layers from the retina to the lobula complex. Firstly, in the retina neural layer, the photoreceptors (R1–R6) are utilized to perceive the change in luminance. Secondly, in the lamina neural layer, the change in luminance is divided into parallel ON and OFF pathways, and the spatial denoising and spatio-temporal LI mechanisms are regarded as the early visual pre-processing, which can suppress environmental noise and improve motion boundaries. Thirdly, in the medulla neural layer, the non-linear instantaneous feedback divisive contrast normalization mechanism is adopted to reduce local contrast sensitivity; furthermore, the parallel contrast pathways are activated. Finally, the parallel motion and contrast pathways converge on the LPTC in the lobula-complex neural layer and the contrast pathway negatively affects the motion pathway to suppress the high-contrast optical flow. Variable contrast in the figure-ground and environmental noise interference are two major factors that affect the stability and robustness of the motion direction of the translating object detection. The proposed bio-inspired model not only has stable and robust performance in variable contrast in the figure-ground, but also has stable and robust performance in the noise background, with its performance to process both simultaneously, which further approximates Drosophila’s visual neural detection capability. Four groups of experiments were adopted to verify the robustness and stability of the proposed bio-inspired model under variable contrast in the figure-ground and environmental noise interference. We have consistently adhered to the study of how Drosophila utilizes a robust, stable, and lightweight visual neural system to process environmental information from a neuroscientific perspective, which can effectively enhance the current ability to build various bio-inspired visual neural models.

## Figures and Tables

**Table 1:** Literature review concerning the spatio-temporal biological mechanisms.

References	Brief Description
[31]	LEI et al. [31] proposed a robust model based on the lobula giant movement detector (LGMD). In the lamina layer of this model, the denoising mechanism is adopted to reduce the ambient noise, which is beneficial for detecting looming objects, but was not validated in the neural circuit based on the LPTC.
[34]	HONG et al. [34] presented a probabilistic LGMD (Prob-LGMD) model. This model incorporates probability into the synaptic connections between multiple layers (not from the summing layer to the grouping layer) to reduce ambient noise, which is beneficial for detecting looming objects, but was not validated in the LPTC-based bio-inspired model.
[35]	ZHENG et al. [35] put forward a binocular-structure LGMD-based (Bi-LGMD) model. In the S layer of this model, the delayed propagation mode is adopted to implement the LI mechanism, which is beneficial for detecting the depth distance of looming objects.
[36]	WANG et al. [36] proposed a feedback model based on the small target motion detector (STMD) against naturally complex backgrounds. In the lamina layer of this model, the inhibition kernel is adopted to implement the LI mechanism, which is beneficial for detecting small moving objects.

**Table 2:** Literature review concerning the bio-inspired motion direction detection models.

References	Brief Description
[8]	CHEN et al. [8] presented a Drosophila-inspired detection model based on the LPTC that integrates a continuous computational layer from the retina to the central complex, which can effectively extract motion salience in complex dynamic environments.
[12]	FRANCESCHINI et al. [12] proposed a two-quadrant detector (TQD) model. First, the input visual signal is divided into ON and OFF visual pathways, and then the motion detection is performed using input combinations of the same-sign signals, i.e., ON–ON and OFF–OFF.
[13]	EICHNER et al. [13] put forward a four-quadrant detector (FQD) model. First, the input visual signal is divided into ON and OFF visual pathways, and then the motion detection is performed using four parallel input combinations, i.e., ON–ON, ON–OFF, OFF–ON, and OFF–OFF.
[14]	CLARK et al. [14] proposed a weighted-four-quadrant detector (WFQD) model, which is optimized based on the FQD. First, the input visual signal is divided into ON and OFF visual pathways, and then the motion detection is performed using four parallel input combinations, i.e., ON–ON, ON–OFF, OFF-ON, and OFF-OFF; furthermore, the two combinations of ON–OFF and OFF–ON are weighted.
[16]	FU et al. [16] presented a motion vision pathway model based on the LPTC of Drosophila, which was used to decode the motion direction of translating objects against complex moving backgrounds; however, the noise environment and model response fluctuation are not considered.
[37]	WANG et al. [37] adopted the max operation algorithm to simulate the physiological mechanism of Tm9 neurons, which is adopted to estimate the motion direction of the translating background, but the model did not validate the motion direction of translating objects against complex dynamic backgrounds.
[38]	XU et al. [38] proposed an improved LPTC-based model, which adopts smooth and threshold filtering to maximize the extraction of valuable motion information against complex dynamic backgrounds; however, the noise environment and model response fluctuation are not considered.
[39]	WIEDERMAN et al. [39] demonstrated that the EMD model [9] is integrated into the elementary small target motion detector for detecting the directional selectivity of small-moving objects, which can detect the four basic translating directions of small-moving objects.

**Table 3:** Literature review concerning the contrast neural computation.

References	Brief Description
[26]	BAHL et al. [26] verified that the parallel ON and OFF contrast pathways start from the medulla, additionally, the parallel ON and OFF contrast pathways negatively affect the parallel ON and OFF motion pathways, which can attenuate the high-contrast signal.
[42]	DREWS et al. [42] demonstrated that the divisive contrast normalization based on fast spatial integration of neural feedback plays a significant role in the visual neural system of Drosophila, which happens prior to motion correlation. Specifically, the foreground signal intensity of each medullary inter-neuron is divided into its neighboring background field via spatially integrating surrounding feedback signals.
[43]	FU et al. [43] proposed a bio-inspired neural network model with contrast vision computation for estimating the natural background motion, but the noise environment is not considered and is not verified in detecting the motion direction of the translating object.
[44]	LI et al. [44] proposed a single LGMD-based bio-inspired pathway model with contrast vision computation for looming perception, but the noise environment is not considered.
[45]	FU et al. [45] presented an ON/OFF LGMD-based bio-inspired pathway model with contrast vision computation, the difference with the reference [44] is that the input visual signal is divided into ON and OFF visual pathways.
[46]	HUA et al. [46] put forward a non-linear bio-inspired pathway model based on lobula plate/lobula columnar type II (LPLC2), which is used to solve the problem of stable collision perception caused by radial motion. Contrast vision computation is applied in the LPLC2.

**Table 4:** The parameter configuration of the proposed bio-inspired model.

Parameters	Description	Value
Δc	A small real number in Equation (3)	0.01
ψ	Baseline contrast sensitivity in Equation (5)	20.0
σ	Standard deviation in the contrast normalization in Equations (6) and (7)	5
sd	Sampling distance in every pairwise detectors in Equations (10) and (11)	4
$\left(\gamma_{1},\;\gamma_{2}\right)$	Exponents of the ON and OFF signals in Equation (12)	(0.5, 0.5)
$\left(W,\;H\right)$	Width and height of two-dimensional visual field in Equation (13)	adaptable

**Table 5:** The experimental results of the inter-quartile range and the sum of the coefficient of variation based on dataset I comparison in Model 1 and the proposed bio-inspired model. Bold indicates the best result.

Indicators	Model 1	Our Model
IQR	8.900 × 10^−10^	**6.228 × 10^−10^**
S	211.2	**121.5**

**Table 6:** The experimental results of the inter-quartile range and the sum of the coefficient of variation based on dataset II comparison in Model 2 and the proposed bio-inspired model. Bold indicates the best result.

Indicators	Model 2	Our Model
IQR	0.283	**0.120**
S	107.5	**88.9**

**Table 7:** The experimental results of the detection success rate (unit: %) based on dataset III comparison for the 6 kinds of bio-inspired models and the proposed bio-inspired model. Text in red, green, and blue are the first, second, and third best results.

Datasets	EMD [9]	TQD [12]	FQD [13]	FU [16]	XU [38]	WANG [50]	Our Model
Dataset III with no noise	99.00	99.03	99.93	99.00	99.90	99.25	99.88
Dataset III with SPN: 0.01	84.68	93.75	94.63	90.23	93.15	91.92	99.70
Dataset III with SPN: 0.02	77.08	90.45	91.18	85.63	92.60	89.03	97.75
Dataset III with SPN: 0.03	69.70	86.33	87.75	80.03	92.38	86.95	94.95
Dataset III with SPN: 0.04	67.25	84.55	86.88	77.53	92.63	75.30	93.33
Dataset III with GN: 10.0	99.00	99.03	99.50	98.98	99.98	99.45	99.85
Dataset III with GN: 20.0	99.03	99.03	99.48	98.98	92.38	99.55	99.80
Dataset III with GN: 30.0	99.03	99.03	99.43	98.88	83.48	99.58	99.83
Dataset III with GN: 50.0	99.03	99.00	99.55	98.95	83.83	99.50	99.63
Dataset III with GN: 80.0	98.95	99.03	99.48	98.80	92.48	99.60	99.83

**Table 8:** The experimental results of the inter-quartile range of the coefficient of variation based on dataset III comparison for the 6 kinds of bio-inspired models and the proposed bio-inspired model. Text in red, green, and blue are the first, second, and third best results.

Datasets	EMD [9]	TQD [12]	FQD [13]	FU [16]	XU [38]	WANG [50]	Our Model
Dataset III with no noise	0	1.321 × 10^−12^	1.806 × 10^−11^	1.787 × 10^−12^	0	0	6.228 × 10^−10^
Dataset III with SPN: 0.01	1.053	0.276	0.196	0.351	0.055	0.859	0.051
Dataset III with SPN: 0.02	2.733	0.536	0.464	0.617	0.073	1.920	0.156
Dataset III with SPN: 0.03	5.414	0.800	0.681	1.172	0.095	3.717	0.276
Dataset III with SPN: 0.04	9.216	1.155	0.948	1.595	0.124	4.541	0.436
Dataset III with GN: 10.0	0.003	0.002	0.002	0.006	0.003	0.003	0.002
Dataset III with GN: 20.0	0.008	0.006	0.003	0.011	0.006	0.007	0.007
Dataset III with GN: 30.0	0.011	0.009	0.005	0.018	0.009	0.009	0.008
Dataset III with GN: 50.0	0.019	0.012	0.010	0.012	0.010	0.012	0.010
Dataset III with GN: 80.0	0.036	0.021	0.016	0.034	0.016	0.019	0.034

**Table 9:** The experimental results of the sum of the coefficient of variation based on dataset III comparison for the 6 kinds of bio-inspired models and the proposed bio-inspired model. Text in red, green, and blue are the first, second, and third best results.

Datasets	EMD [9]	TQD [12]	FQD [13]	FU [16]	XU [38]	WANG [50]	Our Model
Dataset III with no noise	348.3	351.7	357.4	347.9	429.3	336.4	121.5
Dataset III with SPN: 0.01	383.7	349.9	216.9	367.3	437.6	394.6	200.5
Dataset III with SPN: 0.02	565.0	351.8	305.4	341.4	439.3	469.2	218.8
Dataset III with SPN: 0.03	845.3	348.4	331.3	469.9	438.1	508.8	243.0
Dataset III with SPN: 0.04	1178.6	377.0	353.9	593.4	437.4	558.0	266.0
Dataset III with GN: 10.0	344.7	346.7	360.4	346.6	428.6	430.9	125.2
Dataset III with GN: 20.0	344.1	336.5	344.8	344.8	425.0	371.8	126.3
Dataset III with GN: 30.0	343.3	327.6	352.2	342.1	421.5	360.1	127.1
Dataset III with GN: 50.0	334.1	324.3	345.70	331.4	414.8	349.9	126.9
Dataset III with GN: 80.0	313.2	314.7	318.2	323.1	415.3	334.3	124.5

**Table 10:** The experimental results of the detection success rate (unit: %) based on dataset IV, comparing the 6 kinds of bio-inspired models and the proposed bio-inspired model. Text in red, green, and blue are the first, second, and third best results.

Dataset	EMD [9]	TQD [12]	FQD [13]	FU [16]	XU [38]	WANG [50]	Our Model
Dataset IV with no noise	98.01	97.08	97.94	98.06	97.90	98.06	98.65
Dataset IV with SPN: 0.01	83.59	92.65	92.66	90.79	92.19	91.98	97.69
Dataset IV with SPN: 0.02	73.08	90.45	91.18	85.63	92.60	87.36	96.45
Dataset IV with SPN: 0.03	65.45	86.98	85.99	84.65	91.99	84.42	93.99
Dataset IV with SPN: 0.04	56.66	80.84	82.59	82.53	90.11	79.98	92.68
Dataset IV with GN: 10.0	97.89	98.63	98.50	98.01	97.85	97.88	97.97
Dataset IV with GN: 20.0	97.43	97.91	97.48	97.55	90.65	96.98	97.66
Dataset IV with GN: 30.0	97.56	97.09	97.43	97.19	86.33	96.68	97.95
Dataset IV with GN: 50.0	97.49	96.88	97.55	97.23	85.76	96.70	97.78
Dataset IV with GN: 80.0	96.39	97.49	96.48	96.39	84.33	97.00	97.45

**Table 11:** The experimental results of the inter-quartile range of the coefficient of variation based on dataset IV, comparing the 6 kinds of bio-inspired models and the proposed bio-inspired model. Text in red, green, and blue are the first, second, and third best results.

Datasets	EMD [9]	TQD [12]	FQD [13]	FU [16]	XU [38]	WANG [50]	Our Model
Dataset IV with no noise	0.623	0.127	0.240	0.226	0.517	0.489	0.153
Dataset IV with SPN: 0.01	1.188	0.190	0.260	0.313	0.574	0.846	0.233
Dataset IV with SPN: 0.02	2.166	0.272	0.294	0.387	0.589	1.574	0.310
Dataset IV with SPN: 0.03	3.337	0.405	0.366	0.416	0.575	2.034	0.326
Dataset IV with SPN: 0.04	4.629	0.581	0.422	0.458	0.592	2.721	0.367
Dataset IV with GN: 10.0	0.654	0.146	0.255	0.249	0.599	0.500	0.166
Dataset IV with GN: 20.0	0.668	0.176	0.279	0.257	0.654	0.502	0.186
Dataset IV with GN: 30.0	0.705	0.204	0.301	0.279	0.775	0.561	0.200
Dataset IV with GN: 50.0	0.783	0.249	0.326	0.312	0.865	0.598	0.202
Dataset IV with GN: 80.0	0.999	0.305	0.369	0.399	0.955	0.617	0.246

**Table 12:** The experimental results of the sum of the coefficient of variation based on dataset IV, comparing the 6 kinds of bio-inspired models and the proposed bio-inspired model. Text in red, green, and blue are the first, second, and third best results.

Datasets	EMD [9]	TQD [12]	FQD [13]	FU [16]	XU [38]	WANG [50]	Our Model
Dataset IV with no noise	420.0	348.5	422.1	410.4	582.8	399.8	168.3
Dataset IV with SPN: 0.01	445.8	436.7	571.5	535.4	650.1	456.7	200.5
Dataset IV with SPN: 0.02	565.0	436.7	305.4	341.4	439.3	578.6	228.1
Dataset IV with SPN: 0.03	845.3	348.4	331.3	469.9	438.1	754.6	267.5
Dataset IV with SPN: 0.04	1316.3	973.4	675.1	482.0	671.4	984.3	316.7
Dataset IV with GN: 10.0	465.2	426.7	360.4	476.3	602.6	408.9	169.3
Dataset IV with GN: 20.0	542.3	546.1	534.6	568.1	680.3	490.3	172.5
Dataset IV with GN: 30.0	655.5	669.0	718.2	699.7	764.2	646.7	178.2
Dataset IV with GN: 50.0	965.0	936.2	988.7	856.4	939.4	825.3	189.1
Dataset IV with GN: 80.0	1200.4	1187.1	1098.6	1110.6	1165.7	1099.5	225.2

**Table 13:** The experimental results of the inter-quartile range and the sum of the coefficient of variation based on one sub-dataset (SPN ratio: 0.04) of the dataset IV comparison for Models 3, 4, 5, 6, 7, and the proposed bio-inspired model. Text in red, green, and blue are the first, second, and third best results.

Indicators	Model 3	Model 4	Model 5	Model 6	Model 7	Our Model
IQR	1.546	0.560	0.512	0.495	0.361	0.367
S	604.8	425.0	477.6	433.6	320.8	316.7

**Table 14:** The experimental results of the inter-quartile range and the sum of the coefficient of variation based on one sub-dataset (GN standard deviation: 80.0) of the dataset IV comparison for Models 3, 4, 5, 6, 7, and the proposed bio-inspired model. Text in red, green, and blue are the first, second, and third best results.

Indicators	Model 3	Model 4	Model 5	Model 6	Model 7	Our Model
IQR	0.267	0.244	0.240	0.286	0.258	0.246
S	189.8	177.4	190.5	230.5	228.8	225.2

## Data Availability

The code used to generate the results and figures is available in a Github repository, which can be accessed via the following link: https://github.com/szhanghh/Bio-inspired-model, accessed on 6 January 2025. The panoramic real-world dataset is available in a Github repository, which can be accessed via the following link: https://github.com/szhanghh/Model-dataset and https://github.com/szhanghh/Model-dataset2, accessed on 6 January 2025.
